# The Technic of Tubal Sterilization

**Published:** 1913-03

**Authors:** 


The Technic of Tubal Sterilization.—L. W. Sittig, Davenport, Iowa (*Surg., Gyn. and Obs.*, October, 1912). With an extensive reference to the literature, the author reviews the uncertainty of all methods of sterilization of the female, save the cornual excision, which has proved the most certain. This in view of the growing movement for compulsory sterilization of the hopelessly mentally deficient.

